# Acute Brucellosis with a Guillain-Barre Syndrome-Like Presentation: A Case Report and Literature Review

**DOI:** 10.3390/idr13010001

**Published:** 2021-01-01

**Authors:** Ali Alanazi, Sara Al Najjar, Jnadi Madkhali, Yaser Al Malik, Athal Al-Khalaf, Ahmad Alharbi

**Affiliations:** 1 Division of Neurology, King Abdulaziz Medical City, Ministry of the National Guard-Health Affairs, Riyadh 11426, Saudi Arabia; dr.saraalnajjar@yahoo.com (S.A.N.); Jnady888@gmail.com (J.M.); dr.almalik@gmail.com (Y.A.M.); athalalkhalaf@yahoo.com (A.A.-K.); 2 College of Medicine, King Saud bin Abdulaziz University for Health Sciences, Riyadh 14611, Saudi Arabia; 3 Division of Infectious Diseases, King Abdulaziz Medical City, Ministry of the National Guard-Health Affairs, Riyadh 11426, Saudi Arabia; alharbiah3@ngha.med.sa

**Keywords:** brucellosis, neuropathy, GBS, *B. melitensis*, neurobrucellosis

## Abstract

Introduction: Brucellosis is a zoonotic disease that can affect the central and peripheral nervous system and it has variable neurological manifestation. However, brucellosis infection that presents with acute peripheral neuropathy mimicking Guillain-Barre syndrome (GBS) is rarely reported in the literature. Objective and method: We report a 56-year-old man who was initially diagnosed with GBS, and then he was confirmed to have acute *Brucella* infection. We also did a systematic literature review to study the natural history and management of previously reported cases of brucellosis that presented with manifestations consistent with GBS. Results: We found 19 (including our patient) cases of brucellosis that presented with GBS-like manifestations. The age range was 9–62 years. Eight (42.1%) patients had a history of fever. Seven (36.8%) patients had no constitutional symptoms. Five (26.3%) patients had splenomegaly. *Brucella* serological tests were positive in all patients, while blood *Brucella* culture was positive in three (37.5%) out of eight patients. Albuminocytological dissociation was present in nine (64.3%) out of 14 patients. Nerve conduction studies and electromyography were consistent with demyelination polyneuropathy in eight (42.1%) patients, with axonal polyneuropathy in six (31.6) patients, and with mixed axonal and demyelinating polyneuropathy in one (5.3%) patient. Spine MRI showed root enhancement in three (42.9%) patients. Conclusion: In regions endemic with brucellosis, acute peripheral neuropathy presentation may warrant investigations for *Brucella* infection.

## 1. Introduction

Brucellosis is a zoonotic disease that has been reported since ancient times; however, the cause was only discovered in the 19th century. Human brucellosis is caused by four *Brucella* species: *B. melitensis*, *B. abortus, B. suis,* and *B. canis*. Brucellosis infection in humans can present with variable manifestations, including osteoarticular, sacroiliitis, hepatitis, or neurological symptoms [1]. Neurological manifestations occur in 3–5% of patients and can include meningoencephalitis, cerebellitis, myelitis, cranial neuropathy, and peripheral neuropathy [2]. Peripheral nervous system involvement was found in 7% of neurobrucellosis cases [3]; this involvement can be in the form of chronic polyradiculoneuritis or acute neuropathy that can present in a Guillain-Barre syndrome (GBS)-like presentation, but it is rarely reported [4]. 

GBS is a polyradiculoneuropathy that progresses and reaches its nadir within 4 weeks. Clinically, it is characterized by an acute or subacute course, fairly symmetrical weakness, and absent or depressed reflexes, as well as albuminocytologic dissociation in the cerebrospinal fluid (CSF). GBS is believed to be an immune-mediated disease associated with some infectious organisms, such as cytomegalovirus, Epstein-Barr virus, *Campylobacter jejuni,* and *Mycoplasma pneumonia* [5]. However, neuropathy due to neurotropic pathogens can be challenging to differentiate from post-infectious neuropathy secondary to immune-mediated processes like GBS [6]. This may cast doubt on the rarely reported association between GBS and brucellosis. 

Here, we report a 56-year-old man who presented with unsteady gait and lower limb weakness for 1 day. His initial clinical presentation and investigations were consistent with GBS. Later, he was determined to have an active brucellosis infection. In this study, we report the clinical course of our patient and a pooled analysis of 18 previously reported GBS cases with brucellosis. Our aim was to study the clinical course of these cases and to compare it to the natural history of typical GBS cases. 

## 2. Case Report

A 56-year-old man presented to the emergency room with a 1-day history of unsteadiness, walking difficulty, and ascending numbness in the upper and lower limbs. At presentation, he indicated no history of ocular symptoms, facial weakness, bulbar symptoms, upper limb weakness, sphincter dysfunction, or back pain. He also denied any history of fever, night sweats, or recent history of upper respiratory or gastrointestinal infection. However, he had unintentionally lost 7 kg of weight over the month preceding his presentation and this had been associated with generalized fatigue. He also reported contact with sick camels and ingestion of raw camel’s milk. His past surgical history was remarkable for bariatric surgery 2 years previously. 

At presentation, the patient was fully conscious, with fluent speech. Vital signs were stable. His cranial nerve examination was normal. Muscle strength was normal in the upper limbs (UL) and was graded 4 out of 5 in the lower limbs (LL); the LL weakness was slightly worse proximally than distally. His deep tendon reflexes (DTR) were depressed in both the UL and LL. His proprioception sensation was bilaterally impaired in the big toes. 

A lumbar puncture (LP) performed on the second day of admission showed a classic albumin–cytologic dissociation. A nerve conduction study (NCS) and needle electromyography (EMG) revealed evidence of axonal and demyelinating polyneuropathy suggestive of GBS, while a whole spine MRI was unremarkable. Accordingly, he was diagnosed with GBS and started on intravenous immunoglobulins (IVIG) 0.4 g/kg daily for 5 days. 

Over the first week of admission, he progressed gradually and developed asymmetric facial weakness, and his muscle strength was 3 to 4 out of 5 in the UL and 2 out of 5 in the LL. DTR became absent and he developed a stocking-glove distribution of decreased pin prick sensation. In the second week, he was transferred to the rehabilitation ward, where he had some improvement with an intensive rehab program. Ten days later, he went out on a weekend pass and upon his return, his physiotherapist noticed deterioration in his functional mobility in bed. A lumbosacral MRI was done but was unremarkable. A chest X-ray and abdominal and pelvic CTs were conducted to rule out malignancy as the GBS cause, and they were within normal limits. However, his muscle strength continued to deteriorate. He also developed aspiration pneumonia, so he was admitted to the ICU for observation. 

Because of the deterioration after IVIG treatment, we decided to use plasma exchange (PLEX), and he went through seven sessions. Despite a negative initial work up for brucellosis, a repeat *Brucella* serology and blood culture were obtained. Two days later, the *Brucella* blood culture was confirmed as positive and the *Brucella* serology was elevated for *B. melitensis* antibodies. The patient was diagnosed with brucellosis and treated with doxycycline, ciprofloxacin, and streptomycin. After seven sessions of PLEX, his muscle power, as well as his sensory symptoms, showed partial improvement; therefore, he was transferred to the rehabilitation ward again. 

Two weeks later, he deteriorated for a second time, and the muscle strength in his lower limbs was now graded 0 to 2 out of 5. He was transferred back to the neurology ward and received five sessions of PLEX, but without benefit. A third PLEX of five sessions was given 4 weeks later. The CSF study was also repeated at that time and it showed normal results. The patient was referred for the third time to the rehabilitation ward, where he improved consistently and significantly over the next month. He was finally discharged and his power examination upon discharge was graded 4 out of 5 in all limbs. Streptomycin was stopped after 21 days, and he continued on ciprofloxacin and doxycycline for a total of 6 months.

**Figure 1:**
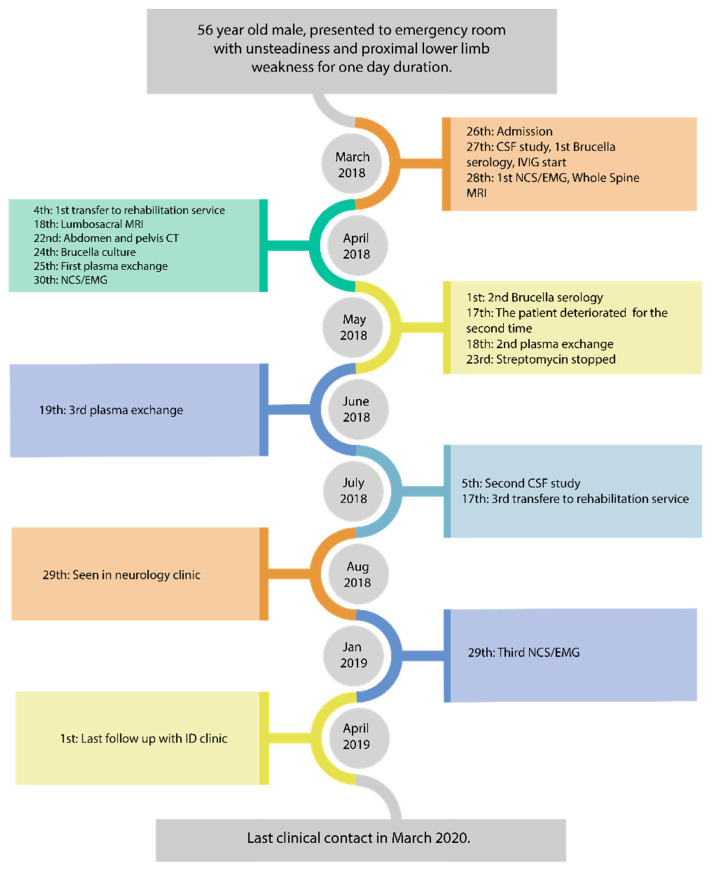
Events sequence for our patient.

In a neurology clinic follow-up 5 months after symptom onset, he was doing fine and had a normal neurology exam, aside from areflexia in the LL. However, his prolonged illness course raised the possibility of chronic inflammatory demyelinating polyneuropathy (CIDP); therefore, the decision was made to administer monthly IVIG for three doses, followed by reassessment. He received the planned doses, but he was lost at follow-up with neurology. He did attend follow-ups with the Infectious Disease clinic; when he was seen one year later, he was walking normally without any signs or symptoms of GBS, so he was discharged from the clinic. The last clinical contact with the patient was two years later by his family physician and the patient was doing well (Figure 1).

### 2.1. Laboratory Investigations

- HgbA1c and blood sugar levels: normal.
- Vitamin B12, B6, B1 and folic acid: normal
- Vitamin E: low (11 mg/L)
- Folate: normal
- Copper: normal
- Immunofixation: normal
- Anti-GM1 ganglioside ant antibodies: negative
- IgG antibodies for EBV and CMV were positive but IgM antibodies were negative.
- *B. melitensis* titer: positive
- 3/27/18: 1/160; 5/1/2018: 1/320; 7/2/2018: 1/640; 11/19/2018: 1:320; 8/23/2019: 1/160.
- *B. abortus* titer: negative throughout the disease course.
- First CSF study: WBC < 1; RBC: 25152; Protein 2.52 g/L; *Brucella* antibody: negative; culture: negative.

### 2.2. Neurophysiological Studies

- First NCS/EMG: showed evidence of mixed axonal and demyelinating polyneuropathy, in keeping with GBS.
- Second NCS/EMG:
  1. Absent superficial peroneal, median, and ulnar sensory responses. The sural sensory response was spared.
  2. Abnormal tibial motor response, with prolonged distal motor latency and reduced conduction velocity in the demyelinating range.
  3. Absent F wave responses for the median, ulnar, tibial, and peroneal nerves.
  4. Needle EMG showed abnormal insertional activity in multiple muscles of the upper and lower limbs, with reduced recruitment and normal motor unit morphology.

These findings were suggestive of subacute, mixed, demyelinating, and axonal polyneuropathy.

- Third NCS/EMG:
  1. Normal sensory responses in the ulnar and sural nerves. Mild prolonged peak latency and reduced conduction velocity in the median sensory response.
  2. Normal motor responses in the tibial, ulnar, and median nerves.
  3. Absent left peroneal motor response.
  4. Normal F wave response for the median, ulnar, and tibial nerves.
  5. Normal needle EMG in multiple muscles of the upper and lower limbs.

### 2.3. Search Strategy

We searched the MEDLINE database and the PubMed engine from inception until February 2020 for the following combination of keywords: “GBS and brucellosis, polyneuropathy and brucellosis, neuropathy and brucellosis, Malta fever and GBS, Malta fever and polyneuropathy, Malta fever and neuropathy, neurobrucellosis, nervous system and brucellosis, nervous system and Malta fever”, looking for all case reports and case series. We found 82 studies. We excluded a total of 47 studies: 27 of those studies described neurobrucellosis presentations other than peripheral neuropathy, 5 studies were written in non-English languages, and 15 studies did not provide sufficient clinical data. 

The remaining 35 studies, which included 53 cases of polyradiculoneuropathy, were extensively reviewed. For our final analysis, we included 18 cases showing an initial presentation that mimics GBS clinical manifestations: acute and subacute relatively symmetrical limb weakness that progressed over days to one month and was associated with hyporeflexia or areflexia. We excluded cases with a decreased level of consciousness, signs of meningeal irritation, spasticity, or an upgoing plantar response. In addition, cases without a clear duration or a pure sensory form of GBS were also eliminated from our final analysis. 

**Figure 2:**
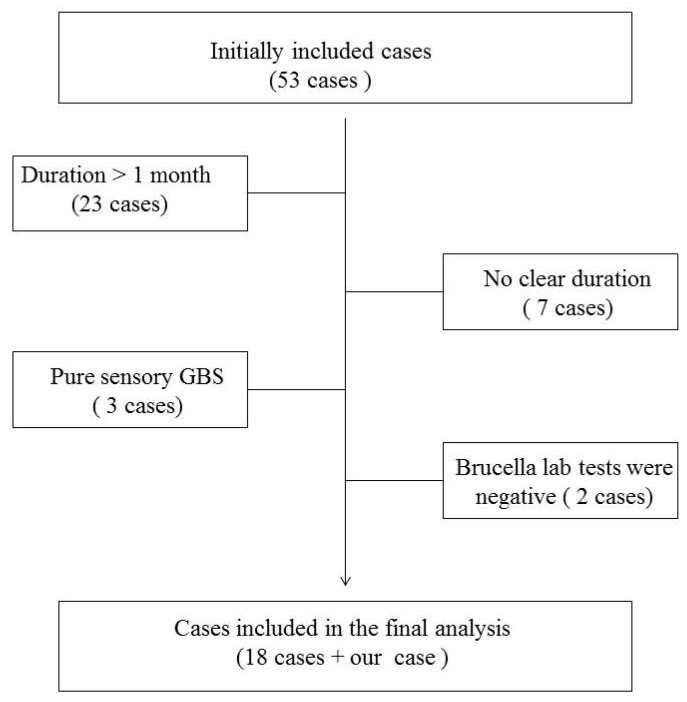
Flowchart demonstrating reasons for cases exclusion from the final anlaysis.

We considered the association between GBS and brucellosis to be present if the clinical suspicion of brucellosis was supported during the active phase of GBS by any of the following laboratory criteria: positive blood culture, positive cerebrospinal fluid (CSF) culture, detection of *Brucella* DNA by polymerase chain reaction (PCR), or positive *Brucella* antibody titer in blood or CSF [7]. Accordingly, we excluded two more cases with a history of brucellosis because the laboratory tests were negative during the active phase of GBS, Figure 2.

## 3. Results

We found 19 cases (including our patient) that fulfilled our criteria. Of the 16 cases where gender was declared, 11 (68.8%) were males. The age range was 9–62 years. Eight (42.1%) patients had a history of fever; three of these had fever associated with night sweats. Five (26.3%) patients had weight loss; one of them had weight loss and night sweats, while seven (36.8%) patients had no constitutional symptoms. Back pain and joint pain were reported by four (21.1%) and two (10.5%) patients, respectively. Splenomegaly was evident in five (26.3%) patients; in one of them, it was associated with hepatomegaly, whereas lymphadenopathy was present in one (5.3%) patient. Five (26.3%) patients had a headache, and facial weakness was also observed in five (26.3%) patients. Ophthalmoplegia and/or diplopia were present in only two (10.5%) patients, while only one (5.3%) patient had speech impairment. Autonomic dysfunction in the form of hypertension and tachycardia was reported in one (5.3%) patient. 

Serological tests for *Brucella* were positive in all patients, but two (10.5%) of them had tests that were initially negative. The blood *Brucella* culture was positive in eight (37.5%) of eight patients. The details of the CSF study were reported for 14 (73.7%) patients and showed lymphocytic pleocytosis in seven (50%) patients, high protein in 100% of the patients, and low glucose in six (42.9%) patients. Albuminocytological dissociation, defined as the presence of high protein with CSF cells <50 cell/μL, was observed in nine (64.3%) patients. *Brucella* antibodies were found in the CSF in eight out of nine patients, while *Brucella* culture from CSF was positive in three out of nine patients. Anti-GM1 ganglioside IgG antibodies were found in one (5.3%) patient. NCS and EMG were consistent with demyelinating polyneuropathy in eight (42.1%) patients, with axonal polyneuropathy in six (31.6%) patients, and with mixed axonal and demyelinating polyneuropathy in one (5.3%) patient. Spine MRI was done in seven patients and showed root enhancement in three (42.9%) patients, mild enhancement in the thoracic region in one (14.3%) patient, and unremarkable results in three (42.9%) patients.

Three patients (15.8%) received IVIG alone, seven (36.8%) received PLEX alone, three (15.8%) received both IVIG and PLEX, and six (31.6%) received neither IVIG nor PLEX. All patients received antibiotics. One (5.3%) patient died and 16 (84.2%) patients regained their ability to walk; the ambulation recovery time ranged from 2 weeks to 1 year (Table 1). 

## 4. Discussion

In the cohort of cases we analyzed, we found some similarities in their natural histories with those of GBS cases. Our case presented with GBS manifestations that evolved initially as a typical GBS case with symmetrical weakness, hyporeflexia, and an absence of both sphincter dysfunction and sensory level. His CSF study showed albuminocytological dissociation, which is consistent with GBS. His NCS and needle EMG also showed acute mixed axonal and demyelination polyneuropathy sparing the sural nerve, which is another characteristic feature of GBS. Nevertheless, the subsequent course of the disease was atypical for GBS and prompted us to repeat the brucellosis test. The results confirm the acuteness of the infection, as the seroconversion happened during hospitalization. The patient reached a nadir after 2 months from the symptom onset, which may suggest an alternative diagnosis such as CIDP. However, NCS and EMG improved and were almost normalized after one year, and the patient has been relapse-free without treatment for 18 months. This could be a monophasic CIDP; however, another possible explanation for this prolonged atypical course is the delay in the treatment of the precipitating infection. 

In our analysis, the majority of the patients were males and approximately two thirds of them reported at least one constitutional symptom. A headache was present in one quarter of the patients. Splenomegaly was the most common organomegaly finding and it was also evident in a quarter of the cases. These clinical findings were described in a pooled analysis of neurobrucellosis cases, with some variation in their frequencies that could be attributed to the small sample in our analysis [3]. However, none of these clinical features were observed in the natural history studies of GBS [23,24].

We found that approximately 10% of the patients had an initial negative *Brucella* serological test; this finding warrants repeating the test if the clinical suspicion is high. Albuminocytological dissociation was present in two thirds of the patients, in agreement with the findings of Fokke et al. [25]. We also noticed a higher frequency of axonal polyneuropathy by NCS and EMG in our analysis in comparison to the previously reported values for GBS cases [23,25]. This discrepancy could be related to a substantially different pathogenesis that causes a brucellosis-associated acute neuropathy/neuronopathy, or it might reflect a different frequency of these findings in GBS cases triggered by brucellosis. A *Brucella* culture from blood or CSF was not sensitive for brucellosis diagnosis, as it was present only in one third of the patients, in conformity with previous studies [2,3]. Approximately one third of the patients did not receive GBS-specific treatment; however, the small number of cases prevented any assessment of whether this had affected the prognosis. 

Brucellosis can cause myelitis and brain-stem encephalitis that, in turn, may mimic GBS [2,6]. This may raise doubts about the certainty of GBS diagnosis in the context of a brucellosis infection. However, the relative symmetry in the motor manifestation and the nearly complete reversibility of the neurological deficits in some cases are consistent with GBS. The presence of demyelinating polyneuropathy in more than one third of the patients is also in agreement with the diagnosis of GBS. Conversely, the protracted course and relapses after GBS treatment in some patients are atypical of GBS. Regardless, we have to bear in mind that brucellosis is a chronic infection; therefore, if it precipitates an autoimmune disease, the autoimmune process may continue as long as the infection remains. Nevertheless, a pure happenstance of GBS in patients with brucellosis cannot be completely ruled out. 

In our literature review, all positive cultures except one grew *B. melitensis.* This *Brucella* species has GM1 ganglioside-like epitopes on its surface, and anti-GM1 ganglioside antibodies have been detected in the serum of mice immunized with *B. melitensis* [26]. Therefore, biological plausibility exists for *Brucella* infection being a precipitator of GBS based on a molecular mimicry hypothesis, as gangliosides are present in the nerve cell membrane [27]. Nonetheless, in our literature review, only one out of three patients tested for anti-GM1 ganglioside IgG showed positive ganglioside anti-GM1 antibodies in the serum. Ganglioside anti-GM1 antibodies are also associated with the acute axonal motor neuropathy variant of GBS and a majority of the cases we found had either demyelinating or sensory motor axonal neuropathy. This may suggest that the GM1-ganglioside antibody is not the only mechanism involved in the pathogenesis of brucellosis-associated, GBS-like neuropathy [28].

However, our analysis is based solely on case reports, which can only partly contribute to general conclusions. Another limiting factor for generalizing this study’s results is that we included only case reports that had been written in the English language. Most of the included case reports also did not provide follow-up data beyond 1 year, which precluded any assessment of the long-term course of this entity. Further prospective research is needed to unravel the natural history of this category of neurobrucellosis. Finally, although a GBS due to brucellosis infection is yet to be confirmed, a GBS-like presentation in areas endemic for brucellosis should alert clinicians to the possibility of brucellosis. This is particularly important if the presentation is associated with clinical features that are atypical for GBS, such as headache, night sweats, organomegaly, or high cell numbers in the CSF. 

## Figures and Tables

**Table 1:** Clinical characteristics of published data of patients with Guillain-Barre syndrome and brucellosis.

First Author	Brucella Culture	MRI Findings	NCS and EMG Findings	Treatment		Prognosis
				Brucellosis	GBS	
Desai [8]	*B. abortus*	NA	NA	DOXY, Streptomycin and TMP/SMX	NA	Complete recovery, after 3 months
Bahemuka *Case # 8* [9]	NA	NA	EMG showed denervation and NCS was normal	RIF and DOXY	NA	Walk with Zimmer frame after 6 weeks
Garcia [10] *(3 cases)* *from abstract and Daoud et al.*	NA	NA	1 case: Pure M axonal PRN 2 cases: M DEM PRN in the remaining 2 patients	RIF and DOXY (4)	PLEX (4)	Unfavorable outcome in one case of axonal GBS (died), partial recovery in the remaining 2 cases of DEM GBS
Al-Eissa [11]	NA	NA	DEM PRN	RIF, DOXY, Streptomycin and TMP/SMX	NA	Completely cured during a 1 year follow up
Hidir [12]	*B. melitensis*	Selective enhancement of ventral lumbosacral nerve roots and cauda equina	DEM PRN with secondary axonal loss	RIF, DOXY, and CFXN	NA	Complete recovery and able to walk unaided after 6 months
Namiduru [13] *from abstract and Daoud et al.*	*B. melitensis*	NA	NA	RIF and TMP/SMX	PLEX (4)	Complete recovery within 1 month
Goktepe [14]	*B. melitensis*	Thickening of the spinal nerve roots and diffuse enhancement along the distal cord and cauda equina	Pure motor PRN with normal sensory NCS	RIF, DOXY, and CFXN	PLEX and IVIG	Able to walk with minimal help after 3 months
Haghighi [15]	NA	Unremarkable	Acute M-S axonal PRN	RIF, DOXY, and CFXN	PLEX and IVIG	Full strength after 1 year
Gul [16] *Case # 8*	*B. melitensis*	Contrast enhancement of lumbar roots	PRN (axonal degeneration)	RIF, DOXY, and CFXN	NA	Barely able to walk unassisted after 6 months.
Shoja [17]	NA	NA	Symmetrical distal axonal S-M PN	RIF and DOXY	N/A “MP 1 g/day for suspected systemic vasculitis”	Weakness resolved after 1 month
Montalvo [18] *From abstract*	NA	NA	Pure M PRN with axonal pattern	RIF and DOXY	PLEX	Complete recovery after 3 months
Aygul [19]	*B. melitensis* (from bone marrow)	Unremarkable	Acute DEM PRN	RIF, DOXY, Streptomycin and TMP/SMX	IVIG	Able to walk unaided after 3 months aside from mild facial diplegia
Babamahmood [20]	NA	Unremarkable	DEM PN	RIF, DOXY, Gentamycin, Streptomycin and TMP/SMX	IVIG	Able to walk normally after 4 weeks
Elzein [21]	NA	NA	DEM S-M PN	RIF, DOXY and Streptomycin	IVIG	Able to mobilize after 3 weeks
Daoud [4]	NA	NA	DEM PRN	RIF and DOXY	PLEX	Partial recovery, and able to walk unaided after 4 weeks
Paydarnia [22]	NA	Mild enhancement of thoracic region	Generalized axonal PN	RIF, DOXY and Ciprofloxacin	PLEX	Walking normally after 6 months

CFXN: ceftriaxone, DEM: demyelinating, DOXY: doxycycline, IVIG: intravenous immunoglobulin, M: motor, MP: methylprednisolone, NA: not available, PLEX: plasma exchange, PN: polyneuropathy, PRN: polyradiculoneuropathy, RIF: rifampicin, S: sensory.
